# A Network-Based Analysis of Disease Complication Associations for Obstetric Disorders in the UK Biobank

**DOI:** 10.3390/jpm11121382

**Published:** 2021-12-17

**Authors:** Vivek Sriram, Yonghyun Nam, Manu Shivakumar, Anurag Verma, Sang-Hyuk Jung, Seung Mi Lee, Dokyoon Kim

**Affiliations:** 1 Department of Biostatistics, Epidemiology and Informatics, Perelman School of Medicine, University of Pennsylvania, Philadelphia, PA 19104, USA; viveksrm@pennmedicine.upenn.edu (V.S.); yonghyun.nam@pennmedicine.upenn.edu (Y.N.); Manu.Shivakumar@pennmedicine.upenn.edu (M.S.); Sanghyuk.Jung@pennmedicine.upenn.edu (S.-H.J.); lbsm@snu.ac.kr (S.M.L.); 2 Genomics and Computational Biology Graduate Group, Perelman School of Medicine, University of Pennsylvania, Philadelphia, PA 19104, USA; 3 Department of Genetics, Perelman School of Medicine, University of Pennsylvania, Philadelphia, PA 19104, USA; anurag.verma@pennmedicine.upenn.edu; 4 Department of Digital Health, SAIHST, Samsung Medical Center, Sungkyunkwan University, Seoul 06351, Korea; 5 Department of Obstetrics and Gynecology, Seoul National University College of Medicine, Seoul 03080, Korea; 6 Institute for Biomedical Informatics, University of Pennsylvania, Philadelphia, PA 19104, USA

**Keywords:** obstetric disorders, pregnancy-related complications, disease–disease network, PheWAS, semi-supervised learning, disease complication, network medicine

## Abstract

Background: Recent studies have found that women with obstetric disorders are at increased risk for a variety of long-term complications. However, the underlying pathophysiology of these connections remains undetermined. A network-based view incorporating knowledge of other diseases and genetic associations will aid our understanding of the role of genetics in pregnancy-related disease complications. Methods: We built a disease–disease network (DDN) using UK Biobank (UKBB) summary data from a phenome-wide association study (PheWAS) to elaborate multiple disease associations. We also constructed egocentric DDNs, where each network focuses on a pregnancy-related disorder and its neighboring diseases. We then applied graph-based semi-supervised learning (GSSL) to translate the connections in the egocentric DDNs to pathologic knowledge. Results: A total of 26 egocentric DDNs were constructed for each pregnancy-related phenotype in the UKBB. Applying GSSL to each DDN, we obtained complication risk scores for additional phenotypes given the pregnancy-related disease of interest. Predictions were validated using co-occurrences derived from UKBB electronic health records. Our proposed method achieved an increase in average area under the receiver operating characteristic curve (AUC) by a factor of 1.35 from 55.0% to 74.4% compared to the use of the full DDN. Conclusion: Egocentric DDNs hold promise as a clinical tool for the network-based identification of potential disease complications for a variety of phenotypes.

## 1. Introduction

With pregnancy-related complication disorders afflicting 8% of the American population, much literature exists regarding acute phenotypes during pregnancy, including preeclampsia, placenta previa, and gestational diabetes [1]. Indeed, it is generally assumed that such pregnancy-related disorders mostly resolve within delivery or shortly afterward [2]. However, recent evidence suggests that women with pregnancy-related disorders are also at risk for long-term medical complications, such as gestational diabetes with type II diabetes, preeclampsia with chronic hypertension and renal failure, and preterm deliveries with cardiovascular disease [2]. As the population of American mothers continues to grow older and develop a higher risk for obesity, the odds of such chronic side effects from obstetric disease only grows greater [3]. Unfortunately, we still do not understand the inner workings of these disease interactions, including the role of genetics [2]. With the lack of research into long-term associations of pregnancy-related diseases, the chronic effects of obstetric disorders remain severely understudied.

**Figure 1:**
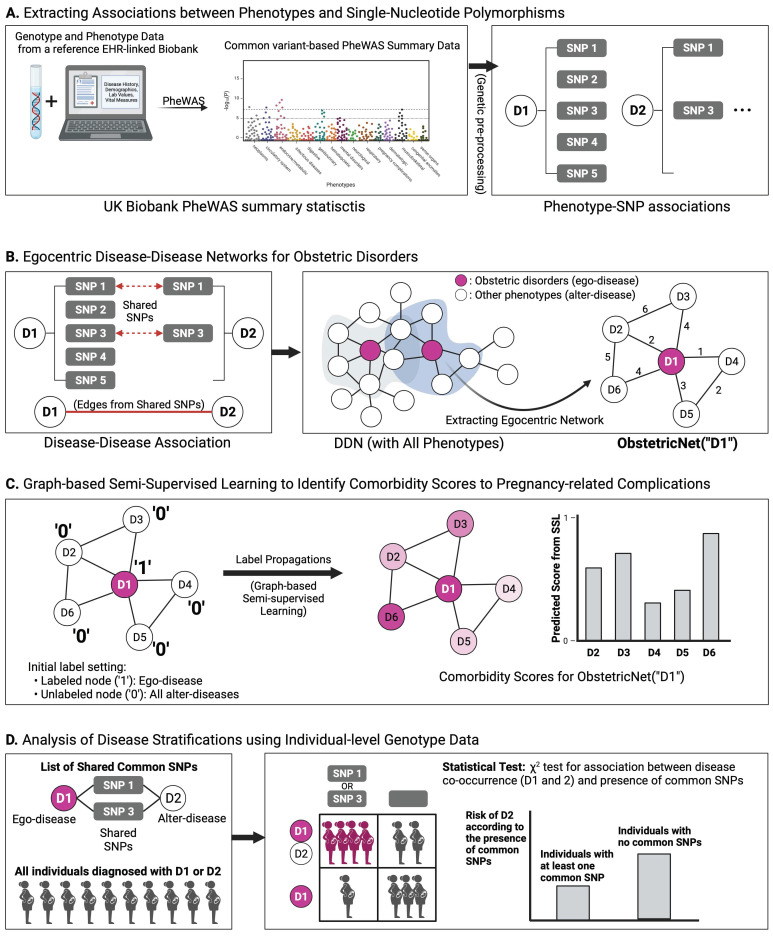
An overview of our pipeline for identifying potential disease complications of obstetric disorders. Using data from PheWAS we can generate a corresponding DDN, where nodes represent diseases and edges represent common associated SNPs between diseases. For each obstetric phenotype of interest, we take a subset of the overarching DDN to generate a corresponding egocentric network, where the central ego-node represents the phenotype in question. All other diseases that share at least one SNP in common with the ego-node are included as alter-nodes in this network. Finally, label propagation through GSSL can be applied to calculate association scores for the alter-nodes, giving us an indication of how related phenotypes are to one another. Individual-level genotype data can be explored to identify how genetic variants for patients might stratify them for comorbidity risk. PheWAS, phenome-wide association study; SNP, single nucleotide polymorphism; DDN, disease–disease network; GSSL, graph-based semi-supervised learning.

The translation from genetic code to cellular activity is a complicated process, involving interactions across organs [4]. Network medicine, the application of graph theory in order to study the connections between diseases, allows us to explore this behavior systematically [4]. In particular, we can represent the full landscape of human disease, the “diseasome”, with a disease–disease network (DDN) [5]. Here, nodes represent phenotypes and edges represent commonalities (such as genetics or lifestyle factors) between phenotypes [5]. A DDN helps visualize the interactions between disorders and subsequent diseases. A network that uses genetic information in its edges can give an indication of potential genetic connections between obstetric diseases and subsequent chronic phenotypes. An effective way to identify genetic associations with phenotypes is with electronic health record (EHR)-linked biobanks. These repositories contain genetic and longitudinal phenotypic data for thousands of patients, including not only DNA samples but also disease histories, laboratory measurements, lifestyle habits, and demographic information [6]. Given an EHR-linked biobank as input, a phenome-wide association study (PheWAS) can be used to calculate a multitude of associations between phenotypes and single nucleotide polymorphisms (SNPs) in an unbiased manner [6]. Using summary statistics from a PheWAS, we can generate a corresponding variant-based DDN, where nodes represent diseases and edges represent shared genetic variants between diseases. Such a DDN can allow us to analyze genetic associations across the diseasome [4]. Given the nature of disease associations between obstetric disorders and their long-term complications, it appears that a holistic, network-based view incorporating knowledge of other traits may help uncover the links between them. In particular, we can use a variant-based DDN to provide insight into the genetic connections between phenotypes. Applied to a DDN, graph-based machine learning methods such as graph-based semi-supervised learning (GSSL) can evaluate the extent of association between a phenotype of interest and other diseases. We start with a source disease node and calculate GSSL scores for all other nodes according to the topology of our network; the higher the score, the more associated the phenotype is with our disorder of interest (Figure 1). Thus, our study applies the concepts of network medicine for the benefit of precision medicine [4]. We aim to be able to use pregnancy developments as a means of identifying patients at high-risk for future disease complications. The variant-based interaction information encoded in our biobank-derived DDN could provide a better understanding of the genetic etiology of pregnancy-related disease complications. The successful completion of this study will produce novel translational results in precision medicine, suggesting possible target variants for follow-up studies of pleiotropy and drug discovery and providing clinical insight into the development of disease complications.

## 2. Materials and Methods

### 2.1. UK Biobank PheWAS Summary Data

We collected UK Biobank PheWAS summary statistics based on EHR-derived broad phenotype codes (PheCodes) obtained from https://www.leelabsg.org/resources (accessed on 16 February 2021) [7,8]. These data correspond to 1403 binary phenotypes for 400,000 British individuals of European ancestry. The Haplotype Reference Consortium panel was used to impute these data, generating 28 million genetic variants [9]. SAIGE (Scalable and Accurate Implementation of GEneralized mixed model), a generalized mixed model association test that handles case-control imbalance, was used to generate summary statistics for each phenotype, providing *p*-values of association between every disease and every variant [10]. These analyses were adjusted for genetic relatedness, sex, birth year, and the first four principal components. Genomic positions are all consistent with Genome Reference Consortium Human genome build 37 (GRCh37). Phenotypes with PheCodes that were specified to the hundredth’s place were excluded from analysis to improve interpretability of the resulting output. To select significantly associated common variants from our data, we performed genetic pre-processing for each phenotype using PLINK (version 1.90, Dr. Shaun Purcell, Center for Human Genetic Research, Boston, MA, USA) and Python (version 3.7, Python Software Foundation, Wilmington, DE, USA) with the following criteria: *p*-value ≤ 1 × 10^−4^, minor allele frequency (MAF) ≥ 0.01, number of cases ≥200, and linkage disequilibrium (LD)-pruning (R^2^ of 0.2 and window size of 250 kilobases) [11]. Finally, given that the focus of our analysis was on female phenotype-wise genetic associations, we excluded all phenotypes in our study that correspond to male-specific diseases, such as prostate cancer (PheCode: 185). Our pre-processing yielded a final set of 697 diseases and 167,556 SNPs. Given these data, we generated a corresponding disease-SNP association matrix, where each element has value ‘1’ if SNPs are significantly associated with disease and value ‘0’ otherwise (Figure 1A). Out of the 697 phenotypes under consideration after filtration, 26 of them corresponded to obstetric disorders. Table 1 includes a few examples of phenotypes under consideration. A full list of all pregnancy-related phenotypes under consideration is detailed in Supplementary Table S1.

### 2.2. Egocentric Disease–Disease Networks for Obstetric Disorders

Using the pre-processed disease–SNP association matrix, we first constructed a complete DDN with 697 phenotypes. This DDN is an undirected, weighted graph G(V,W). As depicted in Figure 1B, nodes V correspond to phenotypes and edges W depict the relationships between diseases. For this network, two diseases are connected to one another if they share at least one associated SNP in common. The weight of the edge between the phenotypes corresponds to the number of shared SNPs between the phenotypes. Although the complete DDN provides insight into the full diseasome, it also includes many phenotypes that are irrelevant as phenotypic associations to pregnancy-related diseases [12]. Thus, obstetric-specific DDNs were constructed to prioritize and predict complications and comorbidities of pregnancy-related diseases based upon the observed genetic associations among multiple phenotypes. Out of the 697 phenotypes in our dataset, 26 of them corresponded to obstetric phenotypes. Thus, we constructed a separate egocentric network for each of the 26 obstetric diseases under consideration to find comorbidity diseases for pregnancy complications [13]. The egocentric network is a specific type of network that focuses on the perspective of a single node (the ego-node) [13]. Each egocentric network is a network in which the single index disease of interest (the ego-disease) is centered, and other diseases (alter-diseases) are directly connected to the ego-disease. We denote our egocentric DDN as ObstetricNet (·), where the value in the parentheses specifies the selected ego-disease. For instance, ObstetricNet (preeclampsia and eclampsia) is an egocentric network centered around preeclampsia and eclampsia. The egocentric DDN is a sub-graph $G^{\prime }\left(V^{\prime },W^{\prime }\right)\subseteq G$ extracted from the complete DDN, where the set of diseases V′ is composed into one ego-disease v^ego^ and other alter-diseases $v_{i}^{\mathrm{alter}}=\;\left\{v_{i}^{\mathrm{alter}}\in V^{\prime }\backslash v_{\mathrm{ego}}\;|\;i=1,\;\ldots ,\left|V^{\prime }\right|-1\right\}$. Alter-diseases are selected if they shared at least one common associated SNP with the ego-disease. As described above, each disease v corresponds to a 167,556-dimensional vector of SNP associations. The similarity W’ was determined by how many SNPs the two diseases share, which was calculated by cosine similarity ($w_{\mathrm{ij}}=\frac{v_{i}\cdot v_{j}}{\left\|v_{i}\|\cdot \|v_{j}\right\|}$). Figure 1B provides an example of an egocentric DDN. In the figure, D1 is an ego-disease and {D2, D3, D4, D5, D6} are alter-diseases.

### 2.3. Network-Based Comorbidity Prediction for Obstetric Disorders

With our 26 distinct egocentric DDNs, a comorbidity scoring algorithm was applied to each network using GSSL [14]. The primary assumption of this approach is that if a pair of phenotypes share more genetic variants, then they will have a higher co-occurrence. For example, we can consider associations of (D1–D3) and (D1–D4) in the egocentric DDN for D1 as shown in Figure 1B. D1 and D3 share four common SNPs, whereas D1 and D4 share just one common SNP. Intuitively, we might expect that D1 and D3 are more likely to co-occur than D1 and D4. Further, when we compare the associations (D1–D2) and (D1–D5), the number of shared SNPs for (D1–D5) is higher than that of (D1–D2). In this case, we may expect that D5 is more likely than D2 to co-occur with D1. However, D2 may also co-occur more with respect to D1 because of the relationship of (D2–D1–D6) and (D2–D1–D3) when we consider the overall underlying structure of DDN.

Considering the full topology of the DDN, we can employ GSSL as a scoring algorithm to predict comorbidities and co-occurrences between phenotypes (Figure 1C). Our scoring procedure and formulation work as follows: suppose that we have an egocentric DDN, $\mathrm{ObstetricNet}\left(\cdot \right)=G^{\prime }\left(V^{\prime },W^{\prime }\right)$, with n diseases (one ego-disease v^ego^ and other alter-diseases $v_{i=1,\ldots ,n-1}^{\mathrm{alter}}$). Let $y={\left(y^{\mathrm{ego}},y_{1}^{\mathrm{alter}},\;\ldots ,y_{n-1}^{\mathrm{alter}}\right)}^{T}$ denote the initial label set and $f={\left(f^{\mathrm{ego}},f_{1}^{\mathrm{alter}},\ldots ,{\;f}_{n-1}^{\mathrm{alter}}\right)}^{T}$ denote the set of scoring results. Since we are mainly interested in the predicted score of the alter-diseases when the ego-disease is given, only y^ego^ is labeled with ‘1’, and the remaining y^alter^ are unlabeled (set to ‘0’) in the initial label set y. The objective of the scoring algorithm for the egocentric network is a function that has the same predicted value as the given label on the ego-disease (labeled node) while also satisfying the weighted average property on the alter-diseases (unlabeled nodes). With this objective, GSSL obtains the predicted score f for the alter-diseases by minimizing the following objective function:

(1) $$\min\;{\left(f-y\right)}^{T}\left(f-y\right)\;+\;{\mu f}^{T}\mathrm{Lf}$$

L=D−W is the graph Laplacian, where $D=\mathrm{diag}\left(d_{i}\right)$ is the diagonal degree matrix and $d_{i}=\sum_{j}w_{\mathrm{ij}}.$ The closed form solution becomes

(2) $$f={\left(I+\mu L\right)}^{-1}y.$$

The resulting score f is transformed with min-max normalization to

(3) $$f^{\prime }=\frac{f-\min\left(f\right)}{\max\left(f\right)-\min\left(f\right)}.$$

As shown in Figure 1C, given an ObstetricNet(‘D1’), the aim of our scoring algorithm is to predict comorbid diseases when patients have an underlying disease (D1). We first initialize label information to apply GSSL. The label for the given underlying disease (D1) is set to ‘1’, whereas the labels for the remaining diseases for which we want to evaluate the comorbidity status (D2~D6) are set to ‘0’. Then, by the closed form solution provided in Equation (2), the initial label on the ego-disease is propagated to the other alter-diseases along the edges of ObstetricNet(‘D1’). The results of this GSSL label propagation provide predicted values for the unlabeled alter-diseases (D2~D6); as an example, the predicted value of D6 (f_D6_) should not be different from the ego-disease (D1) because they shared the most SNPs. The scoring results represent which alter-diseases are most significantly associated with the ego-disease at a genomic level.

### 2.4. Analysis of Disease Stratification Using Individual-Level Genotype Data

As a way of further exploring the effects of genetic associations between diseases, we analyzed individual-level genotype data from an EHR-linked biobank to determine how the variants encoded in our DDN reflect disease co-occurrence patterns in the patient data (Figure 1D). We considered a pair of diseases (the ego-disease and an alter-disease) directly connected to one another in our DDN. The ego-disease v^ego^ and a selected alter-disease v^alter^ consist of 167,556-dimensional SNP vectors, where each vector is composed of binary values, each one standing for the presence (‘1’) or absence (‘0’) of a significant association with a particular SNP. In other words, we can define a set of list SNPs associated with the ego-disease as S^ego^ and a set of SNPs associated with a selected alter-disease as S^alter^. The intersection of the SNP sets $S=\{s_{i}|s_{i}\in S^{\mathrm{ego}}\cap S^{\mathrm{alter}}\;\mathrm{for}\;\forall \;i\}$ represents a list of shared SNPs between v^ego^ and v^alter^. Based on the ego-disease, the alter-disease, and the intersection of the SNP set, we performed the individual-level enrichment analysis on UK Biobank participants. First, we scraped the participants’ phenotype data from our EHR-linked UK Biobank to identify all individuals who ever had both diseases (according to the International Classification of Diseases (ICD)-9 and ICD-10 encodings), as well as all individuals who ever had the phenotype of the ego-node but not the phenotype of the alter-node. To analyze the relationship between the group with variants for shared SNPs in the genotype data as well as the group diagnosed with both the ego-disease and alter-disease in the EHR data, a contingency table was constructed (as shown in Figure 1D). There are two groups: (1) individuals grouped by genetic variants (with/without genetic variants on S) and (2) individuals grouped by clinical records (diagnosed with both v^ego^ and v^alter^ together/diagnosed with only v^ego^). These individuals are scraped from the UK Biobank population as part of the study groups described above. From these two groups of individuals, we then scraped the genotype data of our EHR-linked biobank to identify the subsets of patients who had at least one SNP from the set of SNPs that constitute the edge between the phenotypes. Finally, we performed a chi-squared test for independence on the contingency table. A significant *p*-value from our test suggests that patients can potentially be stratified for increased or reduced comorbidity risk between phenotypes based upon their genetic profiles.

## 3. Results

### 3.1. Network Construction

**Figure 2:**
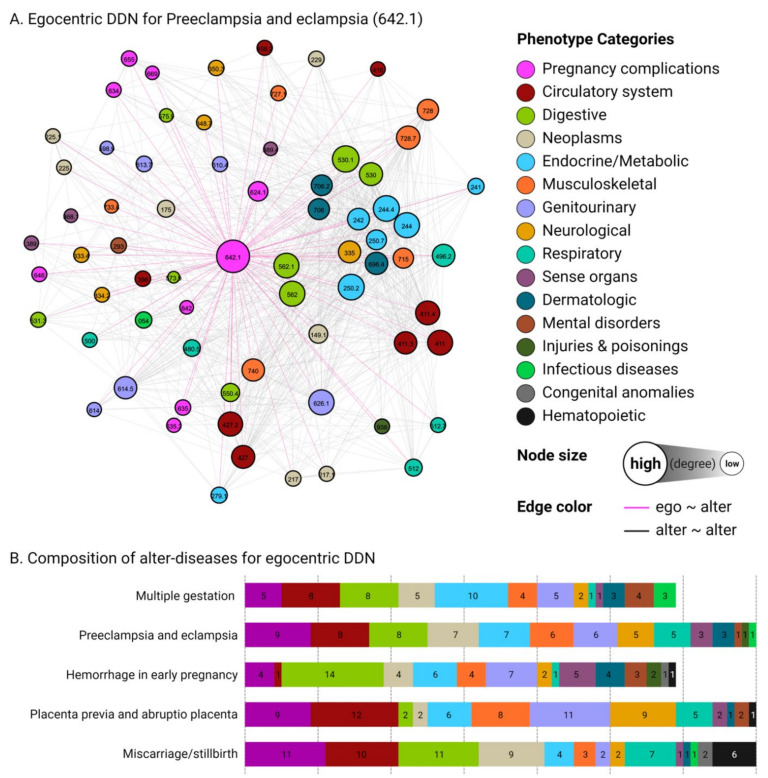
A depiction of ObstetricNet (preeclampsia and eclampsia). (**A**) A visualization of the egocentric network for ObstetricNet (preeclampsia and eclampsia). Nodes are sized by degree and colored by disease category. (**B**) A breakdown of disease categories for the five examples of obstetric disorders under consideration, indicating a wide variety of potential genetic associations between phenotypes.

We constructed 26 egocentric DDNs for each obstetric disorder to observe genetic associations between external phenotypes and selected pregnancy complications. Alter-diseases were included in the egocentric network if they shared at least one associated SNP with the ego-node. Figure 2A depicts ObstetricNet (preeclampsia and eclampsia), the egocentric DDN for preeclampsia and eclampsia (PheCode: 642.1). Preeclampsia is a pregnancy-related hypertensive disorder characterized by liver and kidney damage and has been found to lead to an increased risk of future cardiovascular and metabolic disorders. In this network, the ego-disease (642.1) is located at the center, and other alter-diseases are situated around the ego-disease. The node’s color represents the category of the phenotypes, whereas the node’s size represents the degree, with larger nodes connecting to more neighboring phenotypes. The node for preeclampsia and eclampsia was connected to nine other pregnancy complications, including miscarriage/stillbirth (634), known or suspected fetal abnormality affecting the management of the mother (655), complications of labor and delivery (669), hemorrhage during pregnancy, childbirth, and postpartum complications (635), placenta previa and abruptio placenta (635.3), other complications of pregnancy (646), hypertension complicating pregnancy, childbirth, and the puerperium (642), and dystrophy of the female genital tract (624.1). Through our DDN, we can see that preeclampsia and eclampsia have potential genetic associations with 61 alter-diseases belonging to 13 different disease groups outside of pregnancy complications. For example, we note a connection between preeclampsia/eclampsia and coronary atherosclerosis (411.4), which has been actively researched in terms of long-term outcomes of preeclampsia, as well as a connection with type 2 diabetes (250.2), one of the diseases that can increase the risk of preeclampsia [3,15]. The composition of diseases belonging to the egocentric DDN can be found in Figure 2B, and we provide a full list of alter-diseases for ObstetricNet (preeclampsia and eclampsia) in Supplementary Table S2. The composition of the alter-diseases in Figure 2B demonstrates that obstetric disorders share associated SNPs with a variety of other phenotypes. We proceeded to predict disease co-occurrences with these pregnancy complications based upon this network structure.

### 3.2. Results for Predicting Disease Complications

#### 3.2.1. Generating Ground Truth

To validate and verify the performance of our predictions, we collected and generated ground truth disease co-occurrences from the UK Biobank hospital episode statistic database [16]. Each phenotype was represented by ICD-9 and ICD-10 codes for 502,505 UK Biobank participants. Since our egocentric networks were focused on female-specific traits, electronic health records for 264,796 female participants were used to identify true disease comorbidities. ICD-based diagnostic codes were mapped to PheCodes using the PheCode Map 1.2 (http://phewascatalog.org/, accessed on 17 September 2021) [6]. To calculate disease co-occurrences from EHR data, we used a phi-correlation-based phenotypic disease network proposed by Hidalgo et al. [17]. The Pearson’s correlation for binary variables (Φ-correlation) was calculated for all pairs of diseases out of the 697 phenotypes considered in our PheWAS data. The Φ-correlation for pairs of diseases is expressed as

(4) $$\phi_{\mathrm{ij}}=\frac{C_{\mathrm{ij}}N\;-P_{i}P_{j}}{\sqrt{P_{i}P_{j}\left(N-P_{i}\right)\left(N-P_{j}\right)}},$$

where C_ij_ is the number of patients with both disease i and disease j, and P_i_ and P_j_ are the number of patients with diseases i and j, respectively. A positive value of Φ_ij_ indicates that two phenotypes tend to co-occur, whereas a negative value suggests that the two diseases tend not to co-occur. In our study, the Φ-correlation was calculated between the ego-disease and all alter-diseases belonging to its ego-network. Disease pairs with Φ > 0, *p*-value < 0.05, and C_ij_ > 0 were defined to represent true comorbidity relationships.

#### 3.2.2. Performance Comparison

We applied GSSL to identify diseases at high-risk for future comorbidity or co-occurrence given the onset of pregnancy-related complications. The objective of GSSL for an egocentric network is to sort alter-diseases in order of the strength of genetic association with the selected ego-disease. In order to demonstrate that using the egocentric network is more efficient for identifying disease complications compared to using the full DDN, we performed our experiment with the settings below.

In both the egocentric DDN and the full DDN, only one positive label was assigned to the ego-disease (the chosen pregnancy-related disorder). The rest of the phenotypes in the network all remained unlabeled. Experiments were conducted for our 26 phenotypes of interest. The area under the receiver operating characteristic curve (AUC) and the Spearman’s rank correlation were used to evaluate performance. Table 2 depicts the experimental results for the networks of five selected ego-diseases. Results for all 26 diseases are provided in Supplementary Table S3. The *p*-values derived from the Spearman’s rank correlation demonstrate that ego-networks worked significantly better compared to the full DDN for all 26 phenotypes. When using the egocentric network, the average AUC increased 1.35 times from 55.0% to 74.4% compared to the use of the full DDN. Furthermore, when the rank correlation of the GSSL results was compared to the ground truth disease co-occurrences identified through the Φ-correlation, it was confirmed that the predicted scores of our proposed method were significantly related to the prevalence-based comorbidity measures.

#### 3.2.3. Clinical Implication for Predicted Scores

**Figure 3:**
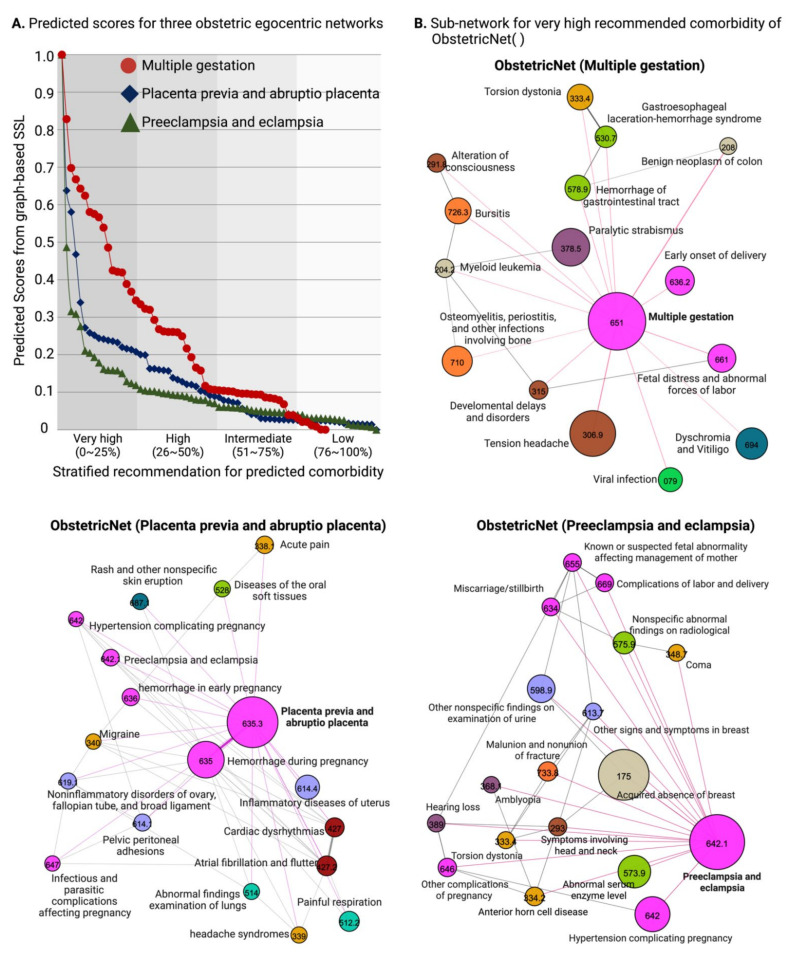
Visualization of the results of our GSSL algorithm. (**A**) An example of disease association scoring curves for placenta previa (PheCode 635.3), multiple gestation (PheCode 651), and preeclampsia (PheCode 642.1). (**B**) Subnetworks depicting phenotypes with scores in the top 25th percentile for each of the three sample phenotypes.

The results in Figure 3 illustrate the output of prediction for various egocentric networks. Here, we provide an example of our GSSL scoring curves (Figure 3A), as well as the sub-networks depicting diseases highly recommended for comorbidity with multiple gestation, preeclampsia and eclampsia, and placenta previa and abruptio placenta. In Figure 3A, three scoring results for ObstreticNet(·) are stacked: ObstetricNet (multiple gestation) marked as red circles, ObstetricNet (placenta previa and abruptio placenta) marked as blue diamonds, and ObstetricNet (preeclampsia and eclampsia) marked as green triangles. Alter-diseases are sorted by the normalized predicted scores transformed by Equation (3). The y-axis depicts normalized predicted scores from GSSL while the x-axis depicts alter-diseases sorted by scores. In the scoring curve, the higher the score of the alter-disease, the higher the chance it has an association with the ego-disease according to the DDN. We stratified alter-diseases by quartiles of predicted scores to prioritize and recommend phenotypes with a high-chance of comorbidity or co-occurrence with the ego-disease. Our groups are defined as follows: very highly recommended group (0th–25th), high (26th–50th), intermediate (51st–75th), and low (76th–100th). Figure 3B depicts the sub-networks for each “very highly recommended” group of the three ObstetricNet (·)’s.

#### 3.2.4. Analysis of Disease Comorbidity Risk Using Individual-Level Genotype Data

The results of our GSSL scoring algorithm provided a unique population-level evaluation of how associated variants between phenotypes may lead to the onset of disease comorbidities. To determine how our network-based conclusions may be applied to individual patients, we performed a proof-of-concept experiment focusing on placenta previa and abruptio placenta (635.3) and hypertensive heart disease (401.2). The two phenotypes share two SNPs in common based upon the input PheWAS summary data. The risk of chronic comorbidity between these phenotypes was compared according to the presence or absence of the shared SNPs among women with our obstetric phenotype of interest, placenta previa. Given these analyses, a chi-squared test for independence yields a *p*-value of 0.0151. In other words, it appears that patients with at least one of the significantly associated SNPs shared between these two phenotypes may be at higher risk for comorbidity between the diseases compared to an individual who does not have one of the two SNPs. This result suggests that the genetic information encoded in our DDNs may be useful for stratifying patients with pregnancy-related disorders into groups at lower or higher risk for comorbidity with other phenotypes.

## 4. Discussion and Conclusions

Recent evidence suggests that pregnancy-related disorders may serve as a window into future disease complications [18]. The associations identified between obstetric diseases and a variety of phenotypes from other disease categories suggest that a network medicine approach may be useful in the identification of disease comorbidities. Indeed, considering a network of diseases may provide clearer insight into disease–disease associations [4]. In particular, a graph that represents genetic variants with its edges might help to identify possible genetic associations with disease co-occurrences.

Here, we developed a network-based scoring algorithm that incorporates a variant-based disease–disease network and GSSL. Our method makes use of the topology of the DDN in order to best predict which phenotypes are likely to be associated with our disease of interest. We considered 26 obstetric disorders and constructed egocentric DDNs for each one using UKBB PheWAS summary data. We then applied GSSL to calculate scores of disease association with our phenotypes of interest. Results were compared to a gold standard of UKBB EHR-derived disease co-occurrences. Our method yielded high AUC and significant Spearman rank correlations when compared to ground truth comorbidities. The use of egocentric DDNs, in particular, boosts our predictive performance compared to the use of the full DDN. Our method revealed some disorders that might play roles as pathogenic mechanisms for obstetric phenotypes, such as inflammatory diseases of the uterus, pelvic peritoneal adhesion, infectious and parasitic complications affecting pregnancy, or noninflammatory disorder of the ovaries, fallopian tubes, and broad ligament [19,20,21]. Furthermore, some disorders were drawn from network analysis that could present after or coincidently with the occurrence of the obstetric phenotype, such as comas and abnormal serum enzyme levels, giving genetic insight into the pathophysiology of obstetric complications [22,23].

As an evaluation of how the conclusions drawn from our network could be applied to individual patients, we considered a single proof-of-concept example between placenta previa and hypertensive heart disease. Analyzing patients who had a SNP shared between the phenotypes as well as those who had co-occurrences of the two diseases suggests that in this instance, the edge information in our DDN can potentially be used to stratify patients who are at high risk of having a comorbidity between the two traits.

A key point to note with our analysis is that the DDN solely represents possible genetic associations between phenotypes; there is no implication of causation. In order to construct a causative DDN, variants from an experiment such as Mendelian randomization would need to be used instead [24]. We also note that the PheCode system of disease classification is imperfect. Thus, conclusions drawn from our analysis need to be considered with this potential inaccuracy in mind. Furthermore, it is important to remember that the genetic profile for the UKBB population may be different from that of the general American population, particularly with respect to genetic diversity. As a result, the conclusions drawn from this dataset may be biased toward a British European perspective. Finally, we use the UKBB to both create our DDN as well as to identify known comorbidities. In spite of the differences in disease definition between our scoring algorithm and our gold standard validation, the fact that we are using the same source dataset in both instances may potentially lead to an overestimation of the accuracy of our results. It would be useful to consider an additional EHR-linked biobank such as the Penn Medicine BioBank [25]. The construction of new DDNs from such datasets, as well as the external validation of our findings with comorbidities from these data, will help verify that our methods work in practice.

In terms of future extensions for our work, the application of DDNs to risk assessment needs to be further evaluated. We will apply our individual-level genotype data analysis to additional pairs of phenotypes. Doing so will help us determine how effective our DDN is at stratifying patients for risk of comorbidity. We will also perform our GSSL scoring on different types of genetics-based DDNs; although this network is based purely on common variants, we can also construct a network according to rare variants or copy number variations. We can even create a polygenic risk score (PRS)-based DDN, where each node includes variants derived from a PRS weight file [26]. Such work will provide further insight into the role of genetics in the links between diseases. It may also be the case that correlations we find between phenotypes are purely incidental in terms of associations with SNPs. It is important that we construct additional DDNs from attributes such as lifestyle factors in order to better understand the nature of disease connection beyond genetics. Finally, the methods we apply here are not limited to obstetric disorders. In the future, we will apply our GSSL approach to score comorbidities for a variety of other disease categories.

## Figures and Tables

**Table 1:** A sample of obstetric disorders under consideration in our analysis.

PheCode	Phenotype Name
634	Miscarriage/stillbirth
635.3	Placenta previa and abruptio placenta
636.3	Hemorrhage in early pregnancy
642.1	Preeclampsia and eclampsia
651	Multiple gestation

**Table 2:** Experimental results of our GSSL scoring algorithm, compared to EHR-derived disease co-occurrences. The use of our method on egocentric networks significantly boosts our ability to identify potential disease comorbidities based upon the genetic information encoded in the DDN. GSSL, graph-based semi-supervised learning; EHR, electronic health record; DDN, disease–disease network; AUC, area under the receiver operating characteristic curve.

Ego-Disease	Egocentric DDN			Full DDN		
	AUC	ρ	*p*-Value	AUC	ρ	*p*-Value
Multiple gestation	0.852	0.272	4.05 × 10^−^^2^	0.526	−0.021	0.531
Preeclampsia and eclampsia	0.823	0.315	8.36 × 10^−^^3^	0.522	0.021	0.534
Hemorrhage in early pregnancy	0.644	0.181	1.75 × 10^−^^1^	0.456	0.044	0.201
Placenta previa and abruption placenta	0.822	0.484	2.94 × 10^−^^5^	0.638	0.129	1.47 × 10^−^^4^
Miscarriage/stillbirth	0.729	0.344	4.13 × 10^−^^3^	0.529	0.030	0.382
Avg. metrics for five selected diseases	0.774	0.319	-	0.534	0.041	-
Avg. metrics for 26 obstetric diseases	0.744	0.210	-	0.550	−7.33 × 10^−^^3^	-

## Data Availability

The UK Biobank PheWAS summary data are publicly available at https://www.leelabsg.org/resources [7]. The UK Biobank individual genotype and electronic health record data were obtained from the UK Biobank (Application Number 68416), and a full list of the variables are available online. These data cannot be shared publicly due to the violation of patient privacy and the absence of informed consent for data sharing. The source code for this study is available upon request from the corresponding authors.

## Supplementary Materials

The following are available online at https://www.mdpi.com/article/10.3390/jpm11121382/s1, Table S1: Obstetric disorders under consideration in our analysis, Table S2: Alter-diseases for ObstetricNet (preeclampsia and eclampsia), sorted by GSSL score, Table S3: Performance results (up to three significant figures) for the graph-based scoring algorithm applied to egocentric DDNs for all 26 obstetric disorders. Rows marked in bold correspond to phenotypes that yielded significant *p*-values compared to a Bonferroni-corrected alpha of 0.05/26 = 1.92 × 10^−3^.
