# Supplementary material for: A Network-Based Analysis of Disease Complication Associations for Obstetric Disorders in the UK Biobank
Source: J Pers Med. 2021 Dec 17;11(12):1382. doi: 10.3390/jpm11121382 (PMC8705804; doi:10.3390/jpm11121382)
Supplement: Supplementary file 1 [file jpm-11-01382-s001.zip › jpm-1479839-supplementary.pdf]

Supplementary Data

Table S1. Obstetric Disorders under consideration in our analysis.

PheCode	Phenotype Name
634	Miscarriage/stillbirth
634.1	Missed abortion/Hydatidiform mole
634.3	Ectopic pregnancy
635	Hemorrhage during pregnancy; childbirth and postpartum
635.2	Antepartum hemorrhage, abruptio placentae, and placenta previa
635.3	Placenta previa and abruptio placenta
636	Early or threatened labor or hemorrhage in early pregnancy
636.2	Early or threatened labor and hemorrhage in early pregnancy
636.3	Hemorrhage in early pregnancy
642	Hypertension complicating pregnancy, childbirth, and the puerperium
642.1	Preeclampsia and eclampsia
643	Excessive vomiting in pregnancy
645	Late pregnancy and failed induction
646	Other complications of pregnancy NEC
647	Infectious and parasitic complications affecting pregnancy
647.1	Infections of genitourinary tract during pregnancy
649	Other conditions or status of the mother complicating pregnancy, childbirth, or the puerperium
650	Normal delivery
651	Multiple gestation
653	Problems associated with amniotic cavity and membranes
655	Known or suspected fetal abnormality affecting management of mother
661	Fetal distress and abnormal forces of labor
663	Umbilical cord complications during labor and delivery
665	Obstetrical/birth trauma
669	Complications of labor and delivery NEC
674	Other complications of the puerperium NEC

Table S2. Alter-diseases for $\mathrm{ObstetricNet}(\mathit{Preeclampsia\ and\ eclampsia})$*, sorted by GSSL score*

PheCode	Phenotype Name
573.9	Abnormal serum enzyme levels
175	Acquired absence of breast
642	Hypertension complicating pregnancy, childbirth, and the puerperium
598.9	Other nonspecific findings on examination of urine

575.9	Nonspecific abnormal findings on radiological and other examination of biliary tract
733.8	Malunion and nonunion of fracture
646	Other complications of pregnancy NEC
348.7	Coma
634	Miscarriage; stillbirth
655	Known or suspected fetal abnormality affecting management of mother
293	Symptoms involving head and neck
389	Hearing loss
669	Complications of labor and delivery NEC
368.1	Amblyopia
500	Lung disease due to external agents
333.4	Torsion dystonia
334.2	Anterior horn cell disease
613.7	Other signs and symptoms in breast
458.2	Iatrogenic hypotension
279.1	Immunity deficiency
350.3	Lack of coordination
610.4	Benign neoplasm of breast
396	Abnormal heart sounds
624.1	Dystrophy of female genital tract
416	Cardiomegaly
054	Herpes simplex
614.5	Inflammatory disease of cervix, vagina, and vulva
531.3	Duodenal ulcer
229	Benign neoplasm of unspecified sites
614	Inflammatory diseases of female pelvic organs
480.5	Bronchopneumonia and lung abscess
149.1	Cancer of oropharynx
938	Dermatitis due to solar radiation
727.1	Synovitis and tenosynovitis
626.1	Irregular menstrual cycle/bleeding
389.4	Tinnitus
740	Osteoarthrosis
550.4	Umbilical hernia
706	Diseases of sebaceous glands
496.2	Chronic bronchitis
250.2	Type 2 diabetes
335	Multiple sclerosis
706.2	Sebaceous cyst
427	Cardiac dysrhythmias
427.2	Atrial fibrillation and flutter
635.3	Placenta previa and abruptio placenta

530.1	Esophagitis, GERD, and related diseases
635	Hemorrhage during pregnancy; childbirth and postpartum
242	Exophthalmos
244.4	Hypothyroidism NOS
696.4	Psoriasis
244	Hypothyroidism
250.7	Diabetic retinopathy
411.3	Angina pectoris
411	Ischemic Heart Disease
715	Other inflammatory spondylopathies
530	Diseases of esophagus
411.4	Coronary atherosclerosis
728.7	Fasciitis
512.7	Shortness of breath
562	Diverticulosis and diverticulitis
562.1	Diverticulosis
728	Disorders of muscle, ligament, and fascia
241	Nontoxic nodular goiter
512	Other symptoms of respiratory system
217	Vascular hamartomas and non-neoplastic navi
217.1	Nevus, non-neoplastic
225.1	Benign neoplasm of brain, cranial nerves, meninges
225	Benign neoplasm of brain and other parts of nervous system

*Table S3. Performance results (up to 3 significant figures) for the graph-based scoring algorithm applied to egocentric DDNs for all 26 obstetric disorders. Rows marked in bold correspond to phenotypes that yielded significant p-values compared to a Bonferroni-corrected alpha of 0.05/26 = 1.92*10^{-3}.*

PheCode	Phenotype Name	Number of Neighboring Nodes	AUC	Rho	P-value
634	Miscarriage/stillbirth	68	0.729	0.343	$4.14 * 10^{-3}$
**634.1**	**Missed abortion/Hydatidiform mole**	**57**	**0.919**	**0.529**	$\mathbf{2.30 * 10^{-5}}$
634.3	Ectopic pregnancy	49	0.550	-0.0524	0.720
635	Hemorrhage during pregnancy; childbirth and postpartum	72	1.00	0.203	$8.77 * 10^{-2}$
635.2	Antepartum hemorrhage, abruptio placentae, and placenta previa	93	0.714	0.196	$5.99 * 10^{-2}$

**635.3**	**Placenta previa and abruptio placenta**	**68**	**0.822**	**0.484**	$\mathbf{2.94 * 10^{-5}}$
636	Early or threatened labor; hemorrhage in early pregnancy	71	0.702	0.270	$2.27 * 10^{-2}$
636.2	Early or threatened labor/hemorrhage in early pregnancy	67	0.825	0.322	$7.95 * 10^{-3}$
636.3	Hemorrhage in early pregnancy	58	0.644	0.181	0.175
642	Hypertension complicating pregnancy, childbirth, and the puerperium	65	0.599	0.106	0.402
642.1	Preeclampsia and eclampsia	69	0.823	0.315	$8.36 * 10^{-3}$
643	Excessive vomiting in pregnancy	42	0.812	0.278	$7.42 * 10^{-2}$
645	Late pregnancy and failed induction	66	0.862	0.153	0.220
646	Other complications of pregnancy NEC	84	0.660	0.232	$3.39 * 10^{-2}$
647	Infectious and parasitic complications affecting pregnancy	75	0.546	-0.0395	0.736
647.1	Infections of genitourinary tract during pregnancy	77	0.686	0.159	0.168
649	Other conditions or status of the mother complicating pregnancy, childbirth, or the puerperium	133	0.584	0.0968	0.268
650	Normal delivery	86	0.618	0.0753	0.491
651	Multiple gestation	57	0.852	0.272	$4.05 * 10^{-2}$
653	Problems associated with amniotic cavity and membranes	86	0.847	0.281	$8.71 * 10^{-3}$
**655**	**Known or suspected fetal abnormality affecting management of mother**	**86**	**0.854**	**0.376**	$\mathbf{3.65 * 10^{-4}}$
661	Fetal distress and abnormal forces of labor	83	0.523	0.0286	0.797
663	Umbilical cord complications during labor and delivery	76	0.753	0.171	0.140
665	Obstetrical/birth trauma	89	0.750	0.248	$1.93 * 10^{-2}$
**669**	**Complications of labor and delivery NEC**	**63**	**0.882**	**0.440**	$\mathbf{3.03 * 10^{-4}}$

674	Other complications of the puerperium NEC	95	0.788	-0.201	$5.12 * 10^{-2}$
